# Preparation of a Sustainable Shape-Stabilized Phase Change Material for Thermal Energy Storage Based on Mg^2+^-Doped CaCO_3_/PEG Composites

**DOI:** 10.3390/nano11071639

**Published:** 2021-06-22

**Authors:** Md. Hasan Zahir, Mohammad Mominur Rahman, Salem K. S. Basamad, Khaled Own Mohaisen, Kashif Irshad, Mohammad Mizanur Rahman, Md. Abdul Aziz, Amjad Ali, Mohammad M. Hossain

**Affiliations:** 1 Interdisciplinary Research Center for Renewable Energy and Power Systems (IRC-REPS), Research Institute, King Fahd University of Petroleum & Minerals (KFUPM), Dhahran 31261, Saudi Arabia; kashif.irshad@kfupm.edu.sa (K.I.); amjad.ali@kfupm.edu.sa (A.A.); 2 Department of Electrical Engineering, King Saud University, Riyadh 11495, Saudi Arabia; momin128@gmail.com; 3 Department of Chemical Engineering, King Fahd University of Petroleum & Minerals (KFUPM), Dhahran 31261, Saudi Arabia; salem.k.basamad@gmail.com (S.K.S.B.); mhossain@kfupm.edu.sa (M.M.H.); 4 Department of Civil and Environmental Engineering, King Fahd University of Petroleum & Minerals (KFUPM), Dhahran 31261, Saudi Arabia; khmohaisen@gmail.com; 5 Interdisciplinary Research Center for Advanced Materials, King Fahd University of Petroleum & Minerals (KFUPM), Dhahran 31261, Saudi Arabia; mrahman@kfupm.edu.sa; 6 Interdisciplinary Research Center for Hydrogen and Energy Storage (IRC-HES), King Fahd University of Petroleum & Minerals (KFUPM), Dhahran 31261, Saudi Arabia; maziz@kfupm.edu.sa; 7 Interdisciplinary Research Center for Refining & Advanced Chemicals (IRC-CRAC), King Fahd University of Petroleum & Minerals (KFUPM), Dhahran 31261, Saudi Arabia

**Keywords:** phase change material, shape-stabilized PCM, high latent heat, little supercooling, thermal energy storage, building comfort

## Abstract

The properties of polyethylene glycol-6000 (PEG)/MgCaCO_3_, a low-cost shape-selective phase change material (ss-PCM), make it highly suitable for solar thermal applications. Nanosized porous MgO-doped CaCO_3_ with Mg molar concentrations of 5%, 10%, and 15% were synthesized using a hydrothermal technique. The prepared MgO-CaCO_3_ matrices were then impregnated with PEG to obtain PEG/MgCaCO_3_ as an ss-PCM. Samples identified as PEG-5MgCaCO_3_ (P-5-MCC), PEG-10MgCaCO_3_ (P-10-MCC), and PEG-15MgCaCO_3_ (P-15-MCC) were prepared and studied. Interestingly, P-10-MCC has the smallest particle size together with a good porous structure compared to the other two materials. The results of thermogravimetric analyses and differential scanning calorimetry indicate that the small particle size and porous structure facilitate the impregnation of approximately 69% of the PEG into the 10-MCC matrix. The latent heat and energy storage efficiency of PEG in the P-10-MCC sample are 152.5 J/g and 96.48%, respectively, which are significantly higher than those of comparable materials. Furthermore, in addition to the improvement of the thermal conductivity of the P-10-MCC, its supercooling is also reduced to some extent. The combined mesoporous and macro-porous structure of P-10-MCC is critical to retaining a large amount of PEG within the matrix, resulting in a high latent heat in the operating temperature range of 35–57 °C. The P-10MCC sample also demonstrates a high energy storage capacity (98.59%), high thermal energy storage/release rates, and exceptional shape-stabilized PCM properties.

## 1. Introduction

In the Middle East, Africa, and South Asia, the cooling demand of buildings dramatically increases from May to August due to the increase of the ambient temperature, with some areas reaching 55 °C. A considerable amount of energy is used for the cooling, heating, air conditioning, lighting, and ventilation of buildings. Recent studies show that buildings consume approximately 40% of the total global energy, which accounts for one-third of the global greenhouse gas emissions [1]. Thus, there is a growing need to drastically reduce the energy consumption by buildings to contribute to the global efforts to minimize effects of greenhouse gas emissions. In regions with hot weather the cooling demand can be significantly reduced by employing renewable phase change materials in building construction. Phase change materials (PCMs) can capture and store solar energy as latent heat during the daytime, which can be used in the night/cooling cycles.

Numerous research initiatives are underway around the world to develop building materials based on low-cost PCMs with the desired properties [2]. The majority of the PCMs studied among the different types of PCMs reported in the literature are hydrated salts and organic paraffin-containing materials [3,4]. However, at high temperatures, the hydrated salts degrade quickly due to loss of some of their water content during each heating/cooling cycle. Organic solids to liquid PCMs, on the other hand, such as those based on polyethylene glycol, paraffin waxes, and fatty acids, are the most widely used materials for heat storage [5,6] due to their ability to self-nucleate and their good thermal reliability [7,8]. However, the major shortcomings of organic PCMs are material loss due to leakage and low conductivity. The leakage issues can be addressed by employing the shape-stabilization of organic PCMs.

Studies are being conducted to investigate expanded graphite (EG), polymers, and metal foam as potential support matrices for producing shape-stabilized PCMs (ss-PCMs) [9,10,11,12]. However, these types of materials have not gained popularity due to their low surface area, low pore volume, and the tedious preparation methods. Porous materials such as MgO [13], CaCO_3_ [14], and SiO_2_ [15] are also being investigated due to their ability to confine PCMs and prevent leakage.

However, published studies indicate that porous materials have not being investigated adequately and/or their energy storage efficiency has not been analyzed thoroughly. In addition, the impregnation ratio (R%), impregnation effectiveness (E%), energy storage capacity (φ%), efficiency of heat storage (γ%), and thermal stability have also not been reported or discussed. Zhang et al. [14] have reported that CaCO_3_ with varying pore sizes effectively decreases the phase separation and the supercooling effect of PCMs. The preparation of the starting precursor calcium oxalate dihydrate, however, is very time-consuming and costly. CaCO_3_ is also widely recognized as a porous support that is non-toxic, of a low cost, and environment friendly. Other research groups have studied the morphology of the porous structure of CaCO_3_ used as a support [16]. Recently, the lead author of this article published interesting results on a PCM based on polyethylene glycol (PEG) using Ca^2+^-doped MgCO_3_ as the support material [17]. Although the X-ray diffraction (XRD) patterns of CaCO_3_ and MgCO_3_ are similar, the intensities and positions of the peaks in the FTIR spectra are quite different from those reported by Xu et al. [18]. TGA analysis of Ca^2+^-doped MgCO_3_ [17] and Mg^2+^-doped CaCO_3_ samples also shows different weight loss profiles. The N_2_ adsorption and desorption isotherms of the two materials show entirely different pore size distributions and pore volumes. The FESEM images are also not comparable. The composition of 5:95 mol % of Mg:Ca (5MgCaCO_3_), 10:90 mol % of Mg:Ca (10MgCaCO_3_), and 15:85 mol % of Mg:Ca (15MgCaCO_3_) of Mg^2+^-doped CaCO_3_ is not the same as that of 5:95 mol % of Ca:Mg (5CaMgCO_3_), 10:90 mol % of Ca:Mg (10CaMgCO_3_) and 15:85 mol % of Ca:Mg (15CaMgCO_3_) of Ca^2+^-doped MgCO_3_, respectively. This implies that CaCO_3_ and MgCO_3_, especially when doped with different metal ions, are not similar compounds. CaCO_3_ has attracted considerable interest as an inorganic support material due to its excellent mechanical properties and favorable thermal conductivity. These properties make CaCO_3_ a better support compared to other inorganic matrices, allowing it to provide higher mechanical protection for PCMs and long-lasting durability [19].

Hence, CaCO_3_ with its porous structure can be an outstanding support matrix for PCMs. Mesalhy et al. [20] found that a porous matrix has a positive effect on the melting/freezing rate of a PCM. Wu and Zhao [21] have demonstrated increased heat storage/transfer capacity of an ss-PCM using metal foams and expanded graphite as the porous support with NaNO_3_ as the PCM. Qian et al. [22] have synthesized an ss-PCM based on PEG with mesoporous calcium silicate as the support, which effectively decreases the solidifying-melting time and the extent of supercooling of pure PEG [22]. However, they have not provided the thermal conductivity, and the reported latent heat value of melting of 110.19 J/g is low. Ma et al. [23] have recently developed a PCM by self-assembling a CaCO_3_ shell and a paraffin core using a surfactant-dependent process, which is time-consuming and costly. Convenient methods for synthesizing porous and fine materials, which can be easily controlled and of a low cost to produce appropriate supports for PEG must be established. PEG has a low vapor pressure and smaller volumetric change in the transition phase [24,25]. Thus, the use of PEG as the PCM and porous CaCO_3_ to produce a shape stabilized composite PCM can provide promising results. PEG-based PCM can also be an excellent solar energy storage material for applications in buildings, notably in hot weather regions, as the operating temperature and other features are highly appropriate. Recently, researchers in the lead author’s laboratory synthesized and reported an octadecanol-MWCNT PCM with a high latent heat and good conductivity [26,27]. However, as the working temperature range is very small, octadecanol-MWCNT PCM is not suitable for applications in buildings. Wang et al. [28] recently developed a PCM by self-assembling a CaCO_3_ shell and a paraffin core using a surfactant-dependent process, which is time-consuming and costly.

In this study, hydrothermal synthesis is used for the preparation of the CaCO_3_ support, as the method provides nanocrystalline porous low-temperature materials at a low-cost [14,17,27]. Also, the hydrothermal method is a low-cost alternative to the sol-gel technique, for which the starting materials are costly. The thermal conductivity of CaCO_3_ can be improved by adding MgO given its higher thermal conductivity compared to CaCO_3_. Thus, MgO doped-CaCO_3_ is expected to serve as an excellent matrix for ss-PCM applications. Porous powders containing Mg^2+^-doped CaCO_3_ were synthesized by optimizing the molar ratio of Ca and Mg nitrates under hydrothermal conditions. A PCM with PEG supported on newly synthesized Mg^2+^-doped CaCO_3_ shows a higher thermal conductivity than PCMs based on paraffin.

## 2. Materials and Methods

### 2.1. Chemicals

Polyethylene glycol (MW 6000) used as the PCM and solvent grade ethanol were purchased from Sigma-Aldrich Co., St. Louis, MO, USA. Ca(NO_3_)_2_·9H_2_O, Mg(NO_3_)_2_·6H_2_O, and CH_3_CH_2_OH were procured from BDH Chemical Co. (NH_4_)_2_CO_3_ was purchased from Merck (Darmstadt, Germany).

### 2.2. Hydrothermal Method

Mg^2+^ containing CaCO_3_ powders were synthesized using the hydrothermal method at lower temperatures. Three Mg-doped CaCO_3_ (MgCaCO_3_) support matrices were synthesized with a Mg concentration of 5 mol %, 10 mol %, and 15 mol %, with the Mg:Ca molar ratios of 5:95, 10:90, and 15:85, respectively. An appropriate amount each of the starting salts was dissolved in deionized water and the required amount of the (NH_4_)_2_CO_3_ solution (4.1 mol cm^–3^) was added to co-precipitate the metal ions. The solution was briskly stirred for twelve hours while maintaining the pH at 8.5. The precursor suspension was transferred into the 500 cm^3^ plastic container of the hydrothermal rector, which is placed in a steel vessel. The hydrothermal reaction was conducted in the closed vessel for 24 h at 200 °C after ensuring that the system was leak free. After the reaction period, the reactor was allowed to cool and stand at room temperature for at least two hours. The final product was washed with ethanol and deionized water three to five times and dried at 120 °C overnight. Three compositions of Mg^2+^-doped CaCO_3_ were synthesized, and the as-synthesized samples were labelled as 5MgCaCO_3_ (5% Mg), 10MgCaCO_3_ (10% Mg), and 15MgCaCO_3_ (15% Mg).

### 2.3. Preparation of Composite PCM

The PEG/MgCaCO_3_ PCM composite was synthesized by dissolving 0.5 g of PEG-6000 and 0.2 g of MgCaCO_3_ in 50 mL of ethanol with stirring for 30 min to mix the organic polymer and inorganic support well and allowing further dissolution by sonication for 30 min. The PCM was isolated by allowing the ethanol to evaporate at 80 °C for 24 h with stirring. Several composite PCMs with the compositions PEG/5MgCaCO_3_ (P-5-MCC), PEG/10MgCaCO_3_ (P-10-MCC), and PEG/15MgCaCO_3_ (P-15-MCC) were synthesized and characterized. Several other composite PCMs with the compositions PEG/20MgCaCO_3_ (P-20-MCC), PEG/25MgCaCO_3_ (P-25-MCC), and PEG/30MgCaCO_3_ (P-30-MCC) were also synthesized for comparison purposes.

The starting materials used in this study are of a comparatively low cost (CaCO_3_: US$ 80/ton; MgCO_3_: US$ 620/ton). Polyethylene glycol used as the PCM is also of a very low cost (US$ 1.85/kg) compared to US$ 60.00 for 100 g of palmityl alcohol commonly used as an organic PCM. One of the best known paraffin based PCMs used for building applications, paraffin eicosane (C20), is 296 Eur for 500 g.

### 2.4. Characterization

The XRD patterns were obtained using a Bruker D8 advance diffractometer system (Berlin, Germany). The operating voltage was kept at 40 kV and the diffractometer device’s current was maintained at 40 mA. The Cu Kα emission with monochromator graphite was at λ = 1.5405 Å. All data were collected at a scan speed of 3 min^–1^ and within the range of 2θ = 10–70°. Fourier transform infrared (FT-IR) spectra were recorded using the KBr pellet technique using a Bruker FT-IR spectroscope (Bruker AXS Analytical X-ray Systems GmbH, Berlin, Germany). Field emission scanning electron microscopy (FESEM: TESCAN LYRA3, Brno, Czech Republic) was used to determine the particle size and the morphology of the products. The images were collected at a 10 kV acceleration voltage. Energy dispersive X-ray spectra (EDS) were obtained using an Oxford Instruments X-mass detector fitted to a Lyra3 TESCAN FESEM (JEOL USA Inc., Peabody, MA, USA). TEM images of the samples were captured using a transmission electron microscope (JEOL Inc., JEM 2011, Peabody, MA, USA) with a 4k × 4k CCD camera (Ultra Scan 400SP, Gatan, Pleasanton, CA, USA) working at 200 kV. The specific surface area, pore diameter, and pore volume of the samples were determined using a NOVA-1200 device (JEOL USA Inc., Peabody, MA, USA). A Tristar II 3020 system was employed to determine the BET surface area.

The powders were evacuated for three hours at 200 °C and the experiments were conducted at a heating rate of 5 °C/min from room temperature to 600 °C under a dry nitrogen atmosphere. The N_2_ adsorption isotherms were obtained using liquid N_2_ at a very low temperature, i.e., −196 °C. The distribution of pore sizes was calculated using the Barrett–Joyner–Halenda isotherm.

A Hitachi U-4100 spectrophotometer was used to record the UV–Vis absorption spectra. The thermogravimetric analysis (TGA) of the samples was performed using a Shimadzu thermal analyzer (Tokyo, Japan, TA-50). The weight loss data were collected using approximately 10 mg of a sample and at a heating rate of 5 °C/min from room temperature to 600 °C under a dry nitrogen flow. X-ray photoelectron spectroscopy (XPS) was used to determine the chemical composition of the samples. In this regard, an ESCALAB-250 (Thermo-VG Scientific, Waltham, Peabody, MA, USA) with Al-Kα radiation (1486.6 eV) was employed. The XPS spectra were recorded at ambient temperature with a pressure of 5 × 10^−10^ mbar maintained in the specimen chamber.

The melting and freezing points and the latent heat of the samples were determined using a DSC-Q2000. DSC data were collected by heating 8.5 mg of sealed samples in an aluminum pan under an Ar gas flow rate of 20 mL/min at a heating rate of 5 °C/min. The thermal conductivity of the powders was determined with circular disk samples using a TCi Conductivity Analyzer, Canada. This equipment uses a modified transient plane source (MTPS) and the measurement method of C-Therm Technologies.

### 2.5. Light-to-Heat Energy Conversion Experiment

The experimental apparatus and protocols used for light-to-thermal conversion are described in reference [29]. For light irradiation, the samples (diameter 5 cm; mass 5.0 g) were placed in a foam container providing heat insulation. A solar power meter was used to determine the intensity of the solar simulator (PlS-SXE300, Beijing, Chang Tuo, China; TES-1333R, TES Electronic Corp, Taipei, Taiwan). A device consisting of a Pt thermocouple, a thermocouple-to-analogue connector (RS-232-RS-485, Instrument Co., Ltd., Jiangsu Suke, Suzhou, China), and a data logger (SK-130RD106062560021A1, Instrument Co., Ltd., Jiangsu Suke, Suzhou, China) were used to capture the temperature-time curve. The P-10-MCC sample was subjected to cycled light irradiation experiments. Then, 5.0 g of the P-10-MCC sample was placed in a weighing vial (R = 2.5 cm) exposed to a light source for light-to-thermal conversion. After 1 (one) hour of irradiation of the sample, the light source was turned off and the sample was allowed to cool to ambient temperature. The cycling experiments were conducted 200 times. A temperature gradient, or thermocline, separates the hot and cold temperature zones.

## 3. Results and Discussion

### 3.1. XRD

**Figure 1:**
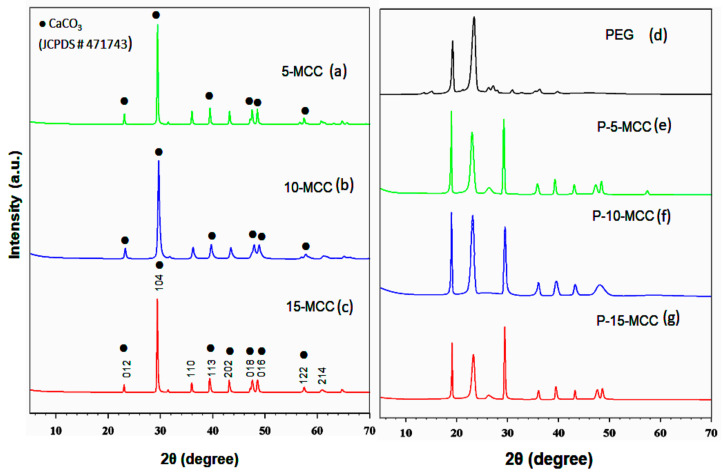
XRD patterns of (**a**) 5-MCC, (**b**) 10-MCC, (**c**) 15-MCC, (**d**) PEG-6000, (**e**) P-5-MCC (**f**) P-10-MCC, and (**g**) P-15-MCC composites.

Figure 1 shows the XRD patterns of the support matrices, (a) 5-MCC, (b) 10-MCC, and (c) 15-MCC used in this investigation. The diffraction peaks at 2θ of 23.1, 29.59, 39.55, 47.9, 48.77, and 57.74 are assigned to calcite planes (012), (104), (113), (018), (016), and (122), respectively. These peaks are assigned to CaCO_3_ in the standard JCPDS file No. 47-1743 [30]. The XRD patterns indicate that all three synthesized samples have the pure calcite structure. However, the peaks of the 10-MCC sample are shifted toward lower diffraction angles. The shift is more significant especially for the peaks corresponding to planes (104), (113), and (116), which is evidence for smaller cations, Mg^2+^ in this case, replacing Ca^2+^ cations [31]. The larger particle size of the synthesized P-20-MCC, P-25-MCC, and P-30-MCC, which is confirmed by FE-SEM, leads to more intense XRD patterns. The latent heat of P-20-MCC, P-25-MCC, and P-30-MCC samples is also low. The XRD patterns of CaCO_3_, MgCO_3_, MnCO_3_, FeCO_3_, and (Mn,Fe)CO_3_ indicate that they have almost the same structure [32]. Nonetheless, substituting Mg^2+^ with Ca^2+^ in MgCO_3_, or Ca^2+^ with Mg^2+^ in CaCO_3_ yields an ordered structure of CaMg(CO_3_)_2,_ which is appropriate for the ionic radii of Ca^2+^ (r = 1.14 A) and Mg^2+^ (r = 0.86 A) [32]. According to Barabas et al. [33], the CaCO_3_ samples formed at a lower concentration of Mg have almost pure calcite structure, and an increase of the Mg/Ca ratio above one causes the crystal lattice structure to change from calcite to aragonite. In this study, the ratio of Mg/Ca is well below one, hence, the crystals remain in the calcite form [22]. Furthermore, Rodriguez-Blanco et al. [34] also found that the stability of amorphous CaCO_3_ (ACC) increases with the presence of Mg, which favors the direct transformation of ACC to calcite and inhibits the crystallization of vaterite.

**Figure 2:**
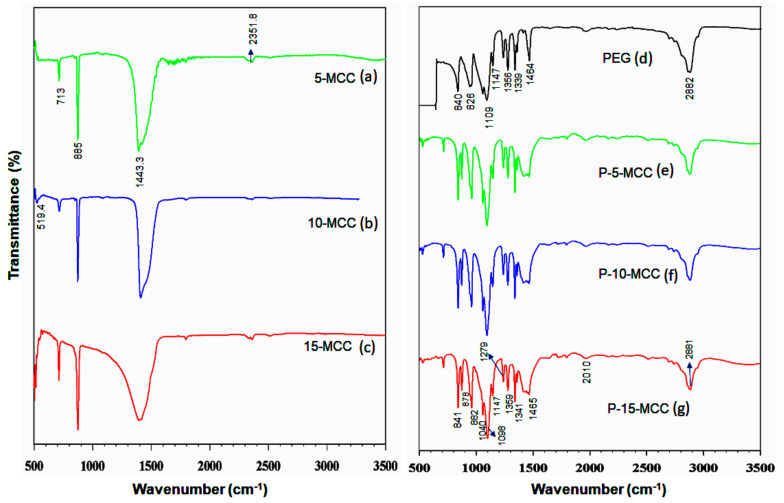
FTIR spectra of (**a**) 5-MCC, (**b**) 10-MCC, (**c**) 15-MCC, (**d**) PEG-6000, (**e**) P-5-MCC (**f**) P-10-MCC, and (**g**) P-15-MCC composites.

**Figure 3:**
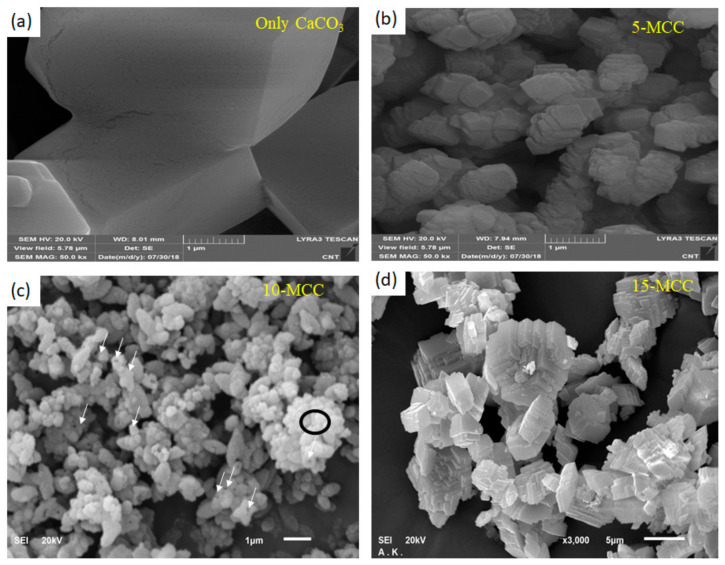
FE-SEM images of the as-synthesized (**a**) CaCO_3_, (**b**) 5-MCC, (**c**) 10-MCC, and (**d**) 15-MCC.

**Figure 4:**
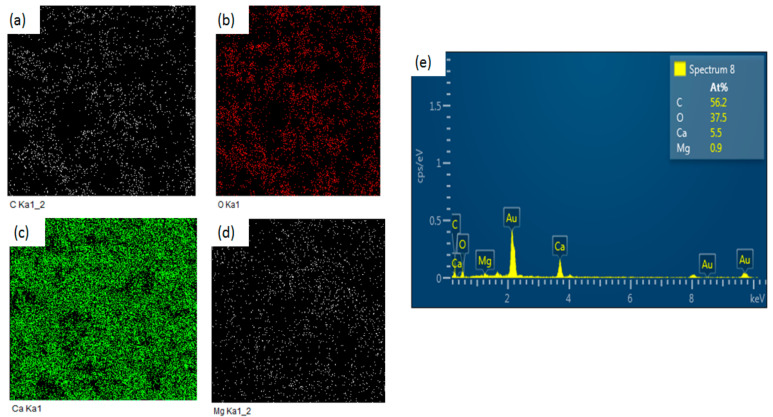
The elemental X-ray mapping images for (**a**) = C, (**b**) = O, (**c**) = Ca, and (**d**) = Mg corresponding to the 10-MCC sample. (**e**) EDS spectrum for the region marked by a circle in 3-1 (**c**).

**Figure 5:**
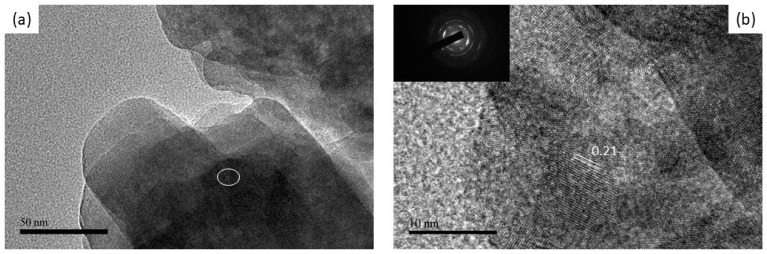
TEM images of (**a**) as-synthesized 10-MCC, and (**b**) and the corresponding HRTEM image with the SAED image in the inset.

**Figure 6:**
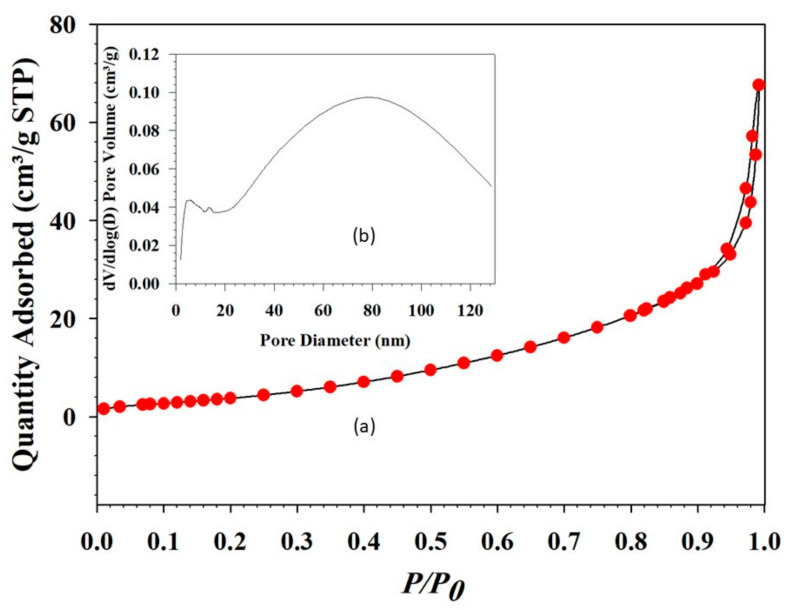
(**a**) Nitrogen adsorption-desorption isotherms of the as-synthesized 10-MCC and (**b**) Inset of the Figure shows the diameter of the pores present in the same sample.

**Figure 7:**
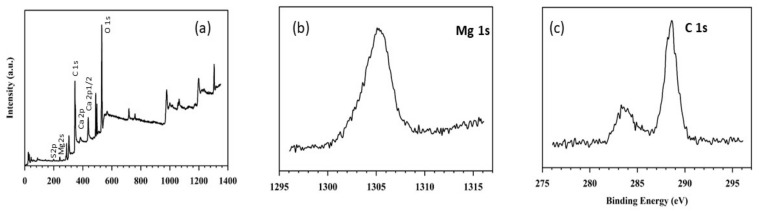

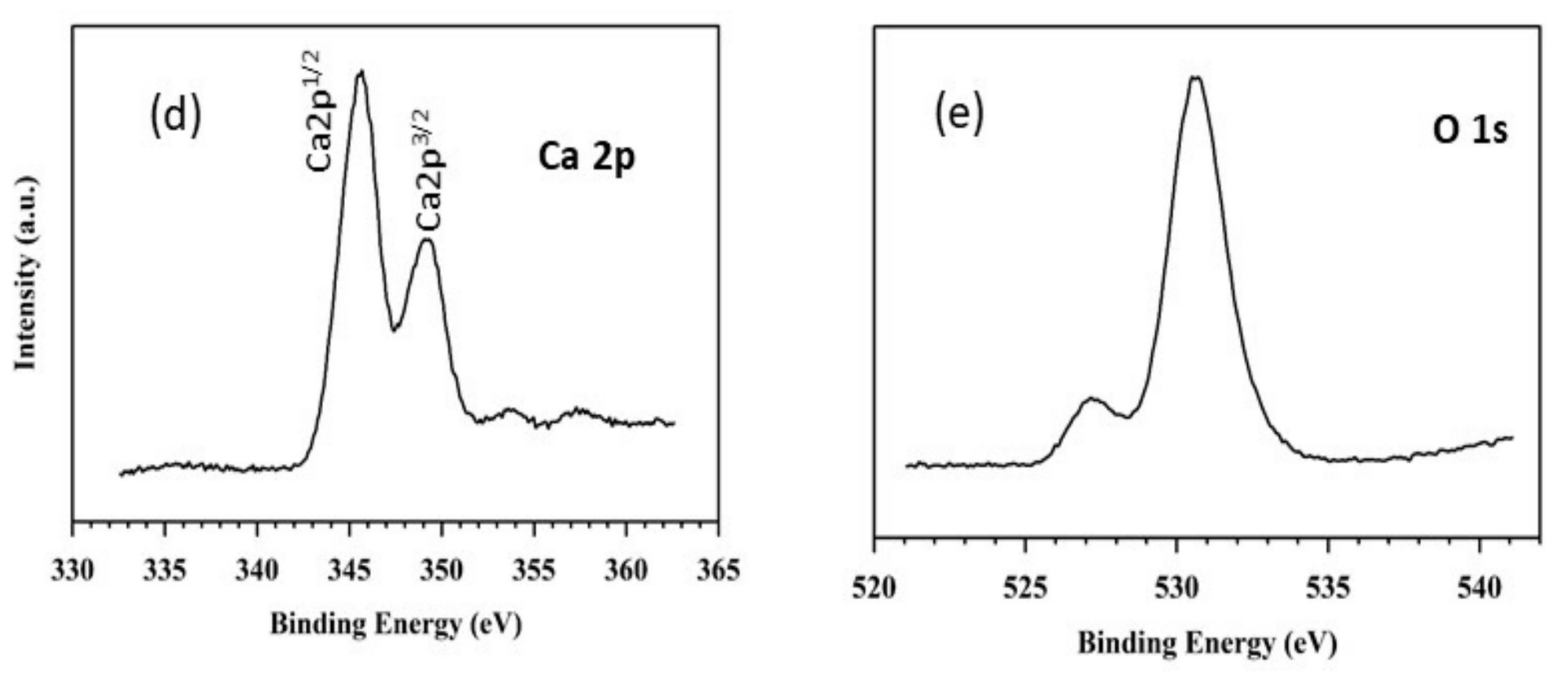
XPS spectra of as-synthesized 10-MCC: (**a**) survey spectrum, (**b**) Mg 1s region; (**c**) C 1s region (**d**) Ca 3p region, and (**e**) O1s region.

**Figure 8:**
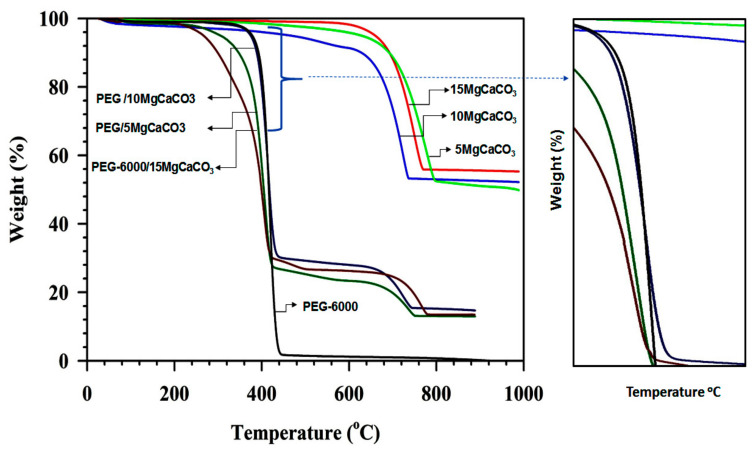
TGA curves of the pure (**a**) PEG, (**b**) 5-MCC, (**c**) 10-MCC, (**d**) 15-MCC, (**e**) P-5-MCC (**f**) P-10-MCC, and (**g**) P-15-MCC composites. The right-side figure is the enlarged part of the left side Figure (marked by arrow).

**Figure 9:**
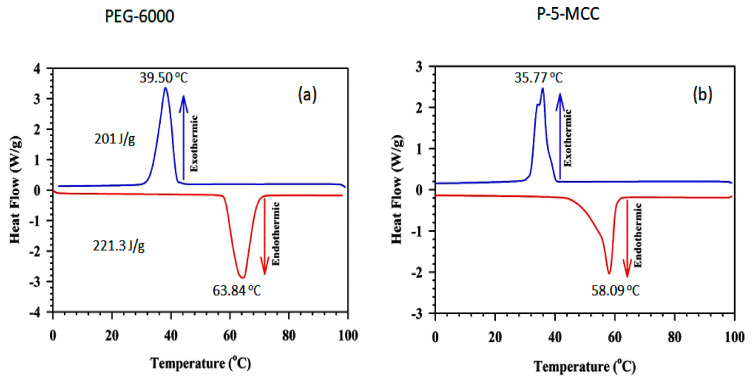
Melting–freezing DSC curves of (**a**) pure PEG and (**b**) P-5-MCC PCM samples.

**Figure 10:**
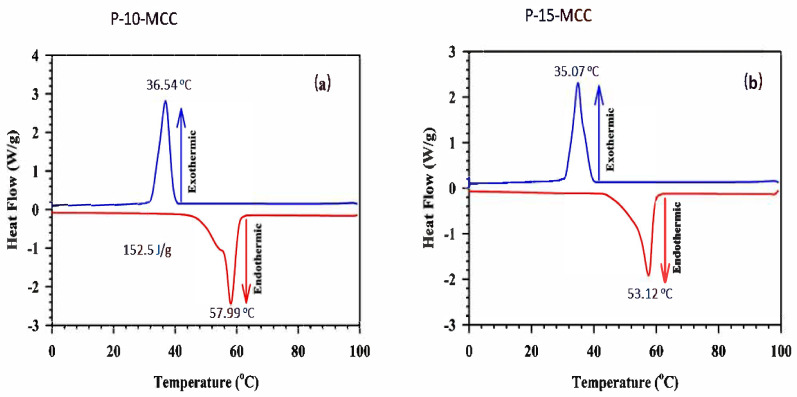
Melting–freezing DSC curves of (**a**) P-10-MCC, and (**b**) P-15-MCC PCM samples.

Figure 1 shows the XRD patterns of the three composites of P-5-MCC (Figure 1e), P-10-MCC (Figure 1f), and P-15-MCC (Figure 1g). For comparison purposes, the XRD pattern of pure PEG-6000 is also shown in Figure 1d. All the composites show the same XRD pattern. The peaks appearing in the 2θ range of 15–30° are similar to the diffraction peaks of crystalline PEG. Intense sharp peaks present at 2θ of 19.24° and 23.42° indicate the presence of crystalline PEG [35]. The XRD patterns of the three composites indicate that PEG and CaCO_3_ exist as a physical mixture without any chemical reactions. The peaks of P-10-MCC and P-15-MCC are smaller than those of PEG alone, indicating that the pores of P-10-MCC and P-15-MCC are occupied by the melted PEG. The occupation of the pores of composites by the PEG melt decreases the crystallite size of PEG. Among the three composites, the XRD pattern of P-10-MCC sample shows the largest decrease in the peak height (Figure 1f). The base of the peaks is also slightly broader. Such XRD patterns were not observed for P-20-MCC, P-25-MCC, and P-30-MCC samples. This indicates that a larger portion of PEG is impregnated into the porous structure of 10-MCC.

### 3.2. FTIR

Figure 2 shows the FTIR patterns of (a) 5-MCC (b) 10-MCC (c) 15-MCC, (d) pure PEG, (e) P-5-MCC (f) P-10-MCC, and (g) P-15-MCC samples. The FTIR bands at wavenumbers of 713.6 cm^−1^, 885.52 cm^−1^, and 1443.23 cm^−1^ are characteristic bands of calcite [30]. Figure 2a has an FTIR band at 713 cm^−1^, which is attributed to the O-C-O in-plane bending vibration of calcite [30]. The FTIR band at a wavenumber of 1443 cm^−1^ is due to ${\mathrm{CO}}_{3}^{2-}$ stretching vibration. The FTIR spectra of all three matrices have bands that match those published for calcite as mentioned above. The FTIR spectra together with the XRD patterns confirm that the synthesized matrices are calcite. The peak at 1109 cm^−1^ of pure PEG shown in Figure 2d is attributed to the stretching vibration of C-O-C [24,30].

The peak at 1095 cm^−1^ is assigned to C-O-H, whereas the one at 1279 cm^−1^ is attributed to OH [35,36,37,38,39]. The two peaks at 1339 cm^−1^ and 1464 cm^−1^ are due to bending vibrations of C-H. The FTIR bands at 2881 cm^−1^ and 2882 cm^−1^ are due to the stretching vibrations of C-H and OH, respectively. The spectra of the composites shown in Figure 2e–g have peaks similar to those of pure PEG (Figure 2d). The peak observed at a wavenumber of 713.6 cm^−1^ in Figure 2a–c is also present in the spectra of the composites shown in Figure 2e–g, indicating that the support matrix remains unchanged. Very intense peaks at 882 cm^−1^ are due to the stretching vibrations of the functional group -CH_2_ [35]. The FTIR spectra of P-10-MCC and P-15-MCC depicted in Figure 2f,g show that peaks of CaCO_3_, as well as PEG, are present. Absence of new peaks indicates that only physical mixing is present resulting in well-mixed CaCO_3_ and PEG composites.

### 3.3. Scanning Electron Microscopy

Figure 3 depicts the FE-SEM images of (a) CaCO_3,_ (b) 5-MCC, (c) 10-MCC, and (d) 15-MCC. Figure 4 shows EDS mapping for 10-MCC. The image of the as-synthesized CaCO_3_ shows that the substance consists of large cubic particles.

The surface of the as-synthesized single-crystal CaCO_3_ has a porous texture, most likely due to hydrothermal synthesis. The doping of CaCO_3_ with 5 mol % Mg^2^ produces multiple layers of smaller porous particles of CaCO_3_ (Figure 3b). Increasing the amount of doping of Mg^2+^ to 10 mol % produces even smaller agglomerated particles as shown in Figure 3c. Among the three Mg^2+^-doped CaCO_3_ matrices, 10MgCaCO_3_ has the smallest particles. At a higher magnification, holes and pores can be observed in the SEM image of 10-MCC. Figure 3d indicates that increasing Mg^2+^ doping further has the opposite effect of increasing the particle size in 15-MCC.

Figure 4 shows the elemental X-ray mapping images of the 10-MCC sample, with (a) = C, (b) = O, (c) = Ca, and (d) = Mg indicating that all the elements are distributed homogeneously. The EDS of 10MgCaCO_3_ depicted in Figure 4e confirms the presence of Mg, Ca, C, and O. The atomic percentages of Ca and Mg in 10-MCC are 5.5 at % and 0.9 at %, respectively. The percentage of Ca with respect to the total concentration of Ca and Mg is 85.9 mol %, and hence the percentage of Mg is 14.1 mol %. These values are not very far from the expected percentages of 90 mol % and 10 mol % of Ca and Mg, respectively. The deviation from the expected values can be due to the non-uniform distribution of the Mg atoms.

Peaks due to gold atoms (Au) are also present in the EDS spectrum due to the application of a gold coating during SEM analysis. The results of the characterization of the three as-synthesized samples using XRD, FTIR, and SEM, indicate that the 10-MCC sample has special properties. Thus, the 10-MCC sample was further characterized using TEM, and the determination of the distribution of pore size and the surface area by BET. The 10-MCC TEM images depicted in Figure 5a show that all the particles are arranged layer by layer. The selected area electron diffraction (SAED) pattern (Figure 5b) was obtained from the layered area of the sample marked with a white circle. The spacing between the fringes of the lattice is 0.210 ± 0.004 nm (Figure 5b). Also, the SAED pattern (Figure 5b) contains non-continuous rings, which suggest that the 10-MCC powders are not crystalline.

### 3.4. Pore Size and Pore Volume

The N_2_ adsorption isotherm of 10-MCC sample shows type IV adsorption and desorption followed by small loops for hysteresis of H3 (Figure 6). The sharp N_2_ adsorption-desorption peak in the high *P/P_0_* range indicates the presence of both mesopores and macropores in the matrix. The calculated BET surface area of the material is 9.5 m^2^ g^−1^ and the pore volume is 0.035 cm^3^ g^−1^. The pore size distribution plots shown in Figure 6b indicate that the porosity of 10-MCC is composed of two types of mesopores, one appearing around 14 nm and a larger pore in the range of 20–110 nm (Figure 6b). The sharp increase in N_2_ adsorption at a relative pressure of 1 (one) shows that macropores exist [40].

The pore size, pore size distribution, pore shape, pore morphology, pore-volume, and specific surface area affect the penetration of supports by polymers. A matrix with very small pores (micropores) is not a suitable medium due to the inability of the liquid polymer to penetrate. Thus, low latent heat values are obtained with such matrices [17]. Macro-porous matrices too are not suitable because the melted PCM cannot be retained within the matrices. However, the absorption of liquid PEG by mesoporous matrices is strongly facilitated by capillary action. A combination of a mesoporous/macro-porous structure support system that can provide a better latent heat value has recently been published [41].

Many ambiguities regarding the behavior of PCMs encapsulated in support material still exist. For example, even though some specific silica [15], ZSM-5 [42], and metal-organic frameworks (MOFs) [43] support materials have a very high surface area, the latent heat of PCMs prepared by mixing them with PEG or paraffin is not very high. However, a typical latent heat value is often demonstrated by materials with a low surface area, such as calcium carbonate, calcium silicate, graphene, and magnesium oxide. More research is required to understand which parameter plays the main role, i.e., pore size or pore-volume, or some other aspect.

An interesting feature of P-10-MCC PCM is the presence of two types of pores as described above, which allow for retaining a substantial amount of PEGs, enhancing the latent heat value of the PCM. Very small pores alter the crystalline structure of a matrix, preventing the material from relaxing to its lowest energy state. Quite large pores are also unacceptable since the melted PCM cannot be retained within the matrix. Mesoporous matrices readily absorb PEG through capillary action, increasing the thermal stability of PEG/10MgCaCO_3_ PCM during melting and freezing cycles, as described later.

### 3.5. XPS

XPS provides the chemical state and composition of a substance. The spectra of as-synthesized 10-MCC sample depicted in Figure 7a, show characteristic peaks due to the position of Mg, Ca, O_2,_ and C on the surface. Two Ca 3d XPS peaks viz., 2p_3/2_ and 2p_1/2_ can be identified at high resolution, due to spin-orbit splitting. According to XPS data the surface concentration of Ca is 1.44 percent. The core level range of Mg 1s is resolved into three component peaks. At a high binding intensity, magnesium hydroxide may have a component peak. Mg is referred to as the height of the second level, and magnesium oxide is related to the peak of the third component. The C-OH and/or O-H groups, and PEG (not shown in Figure 7), are likely to result in peaks at 282, 289.5, and 526, respectively. The presence of an OH group has been reported to likely play an important role in minimizing the supercooling effect [3]. The C 1s range has two peaks as shown in Figure 7c, at 284.2 and 289.3 eV due to C-C. Two O 1s XPS peaks are observed for the as-prepared sample at 527.3 and 531.2 eV (Figure 7e), which is possibly due to hydroxyl group bonding with MgO and CaO. Due to the presence of air and hydrothermal reaction conditions, O peaks at high binding energy are observed. XPS also shows that nitrogen, potassium, or chlorine are absent in the 10-MCC sample, indicating the desired purity of the starting reagents.

### 3.6. Thermal Stability

Figure 8 depicts the TGA profiles of pure PEG (black line), 5-MCC (light green line), 10-MCC (light blue line), 15-MCC (red line), P-5-MCC composite (dark green line), P-10-MCC composite (dark blue line), and P-15-MCC composite (maroon line). The TGA analysis was performed under an atmosphere of argon at a heating rate of 5 °C/min. The results in Figure 8 indicate that the matrices start to decompose into CaO and CO_2_ at higher temperatures near 600 °C. At 800 °C, the weight loss due to the decomposition of the matrices of 5-MCC, 10-MCC, and 15-MCC is 47.55%, 47.02%, and 44.19%, respectively. At about 440 °C, pure PEG starts to decompose, and the PEG decomposition is complete (weight loss is 100%) at about 300 °C. For the composites, the weight loss occurring in the range of 400 °C to 640 °C is most likely due to the removal of the organic molecules present in the composite. According to the weight of the constituents, the composites are expected to contain 28.6% of the matrix and 71.4% of PEG. The weight percentage of these composites remaining at 500 °C are 25.29%, 29.22%, and 26.78% for P-5-MCC, P-10-MCC, and P-15-MCC, respectively. The inorganic porous support matrix of P-10-MCC seems to enhance the thermal stability of PEG by forming a protective shelter. The weight loss percentage of P-10-MCC is 70.78%. The lower degradation temperature of the composites P-5-MCC and P-15-MCC is not well-understood and requires further studies. The fabricated composites possess good thermal stability, and hence, are promising for applications in energy storage systems. The enlarged section of Figure 8 (right side) showing the TGA curves in the temperature range of 400 °C to 480 °C indicates that the slope of the TGA curve for P-10-MCC is slightly lower than that of pure PEG. Thus, in P-10-MCC the rate of heat absorption by PEG is lower, indicating that the 10-MCC matrix inhibits decomposition of encapsulated PEG [37,41].

### 3.7. Differential Scanning Calorimetry

The melting-freezing DSC curves for (a) pure PEG, (b) P-5-MCC, (c) P-10-MCC, and (d) P-15-MCC are shown in Figure 9 and Figure 10. Table 1 shows the DSC results of all samples and comparison with that of different PEG composite PCMs in literature. For the freezing and melting cycles, the enthalpies of pure PEG and the composites were determined using the area under the DSC curves. The pure PEG has a melting enthalpy value of 221.3 J/g, while its freezing enthalpy is 201.0 J/g. Almost the same high enthalpy value has been reported by Liu et al. [44] and Zahir et al. [45].

The composite samples P-5-MCC, P-10-MCC, and P-15-MCC display an apparent melting enthalpy (computed considering the mass of the matrix) [13] or impregnation ratio (R) [22] of 70.94%, 80.43%, and 66.09%, respectively. These results can be explained in terms of the heterogeneity of the composites, in which the mixing and/or penetration of PEG is less than optimal. The validity of the assumption is confirmed by the small peaks of melting and solidification (Figure 9b). For comparison purposes, the DSC data of PEG/mesoporous calcium silicate (MCS) ss-PCM are included in Table 1. The latent heat of the composite denoted ss-CPCM4 matches the highest latent heat achieved for PEG/MCS composites (Table 1). Also, the PEG to support matrix ratio of about 7:3 of the PEG6000/MCS composites is similar to that of the composites used in this study. All parameters were calculated using the standard formulation (see below) [22].

(1) $$R=\frac{\Delta H_{m},\;\mathrm{com}}{\Delta H_{m}\;\mathrm{PCM}}\times 100\%\;$$

(2) $$E=\frac{\Delta H_{m},\;\mathrm{com}+\Delta H_{f},\;\mathrm{com}}{\Delta H_{m,}\;\mathrm{PCM}+\Delta H_{f},\;\mathrm{PCM}}\times 100\%$$

(3) $$\varphi =\frac{\frac{\Delta H_{m},\;\mathrm{com}+\Delta H_{f}\;\mathrm{com}}{R}}{\Delta H_{m,}\;\mathrm{PCM}+\Delta H_{f},\;\mathrm{PCM}}\times 100\%$$

(4) $$\gamma \;=\frac{100\times \Delta H_{m},\;\mathrm{PCM}}{x_{\mathrm{PEG},}\times \Delta H_{m},\;\mathrm{PEG}}$$

In Equations (1)–(4), com = 5-MCC, 10-MCC or 15-MCC support and PCM = support + PEG, where T_m_ = melting temperature, ΔH_m_ = the latent heat in the heating process, T_f_ = freezing temperature, ΔH_f_ = the latent heat in the cooling process, ΔT_s_, = supercooling, X_PEG_ = PEG weight fraction in PCM, R% = impregnation ratio, E(%) = impregnation efficiency, φ% = Energy storage ability (capability), and γ% = heat storage efficiency. The experimental thermal storage efficiency was also calculated using Equation (4) [46,47].

**Table 1:** DSC results of pure PEG-6000 (PEG) and PEG/5MgCaCO_3_, PEG/10MgCaCO_3_, and PEG/15MgCaCO_3_ composite PCMs and comparison with those of different PEG composite PCMs reported in the literature.

Sample	T_f_ (°C)	T_m_ (°C)	ΔH_f_ (J/g)	ΔH_m_ (J/g)	ΔT_s_ (°C)	R%	E%	φ	γ	Reference
* PEG	39.5	63.84	201	221.3	24.3	-	-	-	100.0	This work
* P-5-MCC	35.77	54.36	116.3	134.5	18.59	60.78	59.39	97.72	85.09	This work
* P-10-MCC	36.54	55.17	134.4	152.5	18.63	68.91	67.94	98.59	96.48	This work
* P-15-MCC	35.07	53.12	107.1	125.3	18.05	56.62	55.03	97.20	35.07	This work
PEG1000/MgO	8.30	34.4	-	61.62	16.1	64.6	-	-	-	[13]
PEG10000/SiO_2_	-	61.61	-	162.9	-	-				[15]
PEG1000/SiO_2_-β-AIN	45.13	60.41	161.4	132.9	15.28	-	-	-	-	[37]
PEG6000/CaO_4_Si	44.10	57.03	106.8	122.1	-	-	-	-	-	[22]
PEG	42.02	56.89	176.36	190.08	14.87	-	-	-	-	[48]
PEG/EP	46.33	58.41	137.32	145.14	12.08	-	76.36	77.08	100.95	[48]
PEG/EP/Carbon	46.71	55.19	129.27	134.93	8.48	-	70.99	72.10	101.57	[48]
PEG6000	36.2	61.8	187.1	185.3	25.6	-	-	-	-	[49]
GNS	37.3	60.5	178.8	176.9	23.2	-	95.47	95.52	100.05	[49]
Ag–GNS/PEG-1	35.9	60.2	179.4	177.2	24.3	-	95.63	95.76	100.13	[49]
Ag–GNS/PEG-2	35.6	60.3	175.8	173.3	24.7	-	93.52	93.74	100.23	[49]
Ag–GNS/PEG-3	36	60.2	171.9	169.6	24.2	-	91.53	91.70	100.19	[49]
Ag–GNS/PEG-4	36.1	60.3	167.8	166.1	24.2	-	89.64	89.66	100.03	[49]
Paraffin wax (RT27)	25	25.1	-	154	0.1	-	-	-	-	[50]
RT27/Expanded perlite	25.5	26.3		84	0.8	-	54.55	-	-	[50]
RT27/EP/Sikalatex(SL)	25.8	26.3		51.6	0.5	-	33.51	-	-	[50]
RT27/EP/SL/AL	25.3	26.1		50	0.8	-	32.47	-	-	[50]
Paraffin	23.04	26.83	135.8	136.2	3.79	-	-	-	-	[51]
P1 (EP/Paraffin 20%)	22.62	27.5	11.3	10.4	4.88	-	7.64	7.98	-	[51]
P1 (EP/Paraffin 40%)	22.36	27.34	51.6	53.5	4.98	-	39.28	38.64	-	[51]
P1 (EP/Paraffin 60%)	22.52	27.56	80.8	80.9	5.04	-	59.40	59.45	-	[51]
P1 (EP/Paraffin 80%)	22.38	27.38	118	118.2	5	-	86.78	86.84	-	[51]
Eicosane (C20)	32.92	36.18	268.13	275.91	3.26	-	-	-	-	[52]
EP/C20 60%	34.32	36.12	155.26	161.18	1.8	-	58.42	58.16	-	[52]
EP/C20 60%/CNT 0.3%	34.32	36.24	155.26	160.38	1.92	-	58.13	58.02	-	[52]
EP/C20 60%/CNT 0.5%	35.55	36.34	145.92	159.02	0.79	-	57.63	56.05	-	[52]
EP/C20 60%/CNT 1%	35.53	36.47	141.15	157.43	0.94	-	57.06	54.88	-	[52]

All-star (*) represents the present study, and (-) = Data not available. The maximal deviation determined for phase change temperature and latent heat are ±0.11 °C and ±0.43 J/g, respectively, taking into account the averages of three measurements.

Table 1 compares the characteristics of the materials developed for the present study with those of similar ss-PCMs materials reported in the literature. The results clearly demonstrate that the material developed in the present study possesses favorable thermal characteristics, indicating its potential application for providing comfort within buildings. Even though there are organic and/or inorganic PCMs with a high latent heat, the present study focused on the paraffin and PEG-based PCMs owing to their favorable working temperature range for applications in building. The results indicate that P-10-MCC performs slightly better with an impregnation ratio of 68.9% compared to 66.7% for ss-CPCM4, while the impregnation efficiency (E%) and the energy storage capability (φ%) of P-10-MCC are also higher. The impregnation ratio of P-10-MCC (68.91%) was calculated according to the formula in [22].

Among the tested samples, a high energy storage efficiency of 96.48% was obtained, which is an important parameter in the evaluation of the activity of a PCM. Most of the ss-PCMs reported in the literature do not provide such a high energy storage efficiency (Table 1). The heat storage capability of all the composites evaluated in this study is in the range of 97–98%, with P-10-MCC possessing the highest efficiency and P-15-MCC the lowest (Figure 10a,b, and Table 1). A high energy storage capability of 99.17% was also achieved. The high thermal storage capacity of P-10-MCC composites indicates that almost all PEG molecules efficiently release/store energy through phase transition. A noteworthy aspect is that the energy storage capacity of most of the ss-PCM-based systems obtained using Equation (3) is above 100 as shown in Table 1.

Supercooling (ΔT_s_) were of pure PEG, P-5-MCC, P-10-MCC, and P-15-MCC is 24.34 °C, 18.59 °C, 18.63 °C, and 18.05 °C, respectively. The largest decrease of supercooling of 23.5% was observed for P-10-MCC (Figure 10a). Supercooling can be significantly reduced by increasing the thermal conductivity of a system [3]. The porous structure of ZSM-5 functions as a support matrix, while the interaction between PEG and ZSM-5 affects the melting and solidification temperatures [3]. Zhang et al. have also found that nanosized porous matrix materials can be used to minimize the supercooling effect in PCM systems [14].

**Figure 11:**
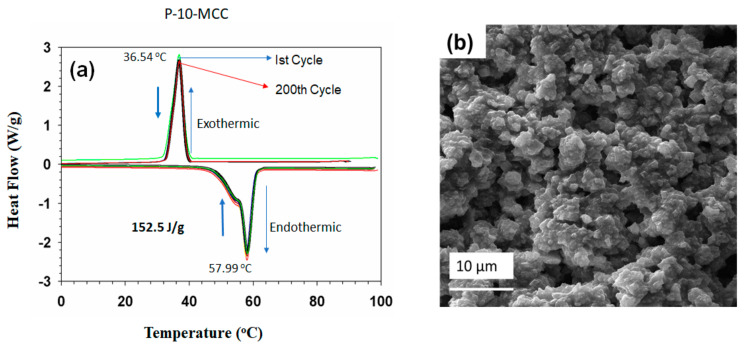
(**a**) Melting–freezing DSC cycling curves of P-10-MCC PCM samples and (**b**) the FE-SEM image, repeated 200 times.

The thermal cycling performance of the composite P-10-MCC PCM, which is important for its commercial feasibility, was also evaluated. P-10-MCC PCM was thermally stable even after recycling 200 times with a DSC curve recorded every twenty cycles. The 1st and the 200th iterations of the DSC cycling of P-10-MCC are depicted in Figure 11a. Both exothermal and endothermal curves do not significantly change with cycling, indicating that the composite has a good life cycle with excellent thermal reliability. This finding shows that PEG can sustain virtually constant phase changes in terms of temperature and enthalpy during multicycle DSC scans. This system can be used to store and release latent heat at a constant temperature under repeated cycling. Even after 200 cycles the sample maintains its original structure, as shown in the FE-SEM image (Figure 11b). The thermal stability of the PCM indicates that liquid PEG has penetrated the matrix by capillary action and surface tension effects. Even though micro- or nano-size pores may hinder penetration by PEG chains, mesoporous/macro-porous structures facilitate the penetration by PEG.

As demonstrated above, doping 5 mol % of Mg^2^ into CaCO_3_ produces multiple layers of smaller particles of CaCO_3_ (Figure 4b). Among the three Mg^2+^-doped CaCO_3_ matrices, 10-MCC has the smallest particles. Figure 4d indicates that increasing Mg^2+^ doping further has the opposite effect with the particles getting larger. An interesting feature of P-10-MCC PCM is the existence of two types of pore structures described above, which can play a crucial role in enhancing the latent heat of the PCM by retaining a substantial amount of PEG. Very small pores change the crystalline nature of a matrix. Hence, probably the substance may be facing a problem in relaxing to its lowest state of energy.

The penetration of PEG into nonporous materials is insignificant, resulting in a low latent heat. A mesoporous structure promotes the capillary uptake of PEG, which provides a higher latent heat value. P-15-MCC PCM has agglomerated particles with an almost non-porous and/or macroporous structure. P-15-MCC PCM is not able to retain liquid PEG, resulting in a low latent heat. Besides, the peaks in the XRD pattern of P-10-MCC (Figure 1f) show the largest decrease in peak height among the three PCM composites. This indicates that a larger portion of PEG is impregnated into the porous structure of 10-MCC compared to 5-MCC and 15-MCC PCM samples. Hence, P-10-MCC shows higher melting and solidification latent heat values. Furthermore, it is important to note that during the melting cycle any vapor/gas formation is not observed. Also, voids are not formed during the freezing process.

### 3.8. Compatibility of the PCM with Metals Used for Containers

**Figure 12:**
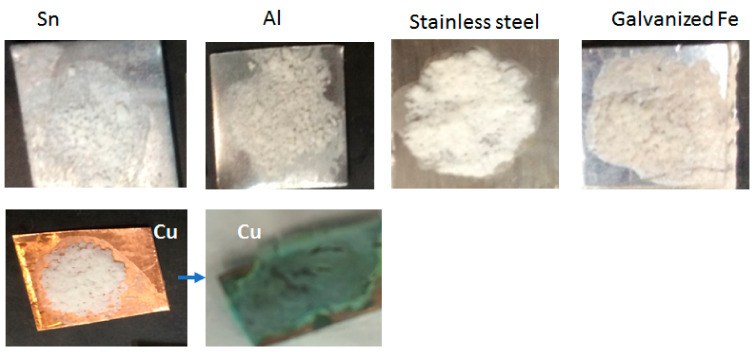
Photographs of the specimens used for the compatibility tests of Sn, Al, Stainless steel, Galvanized Fe, and Cu metal sheets coated with the P-10-MCC PCM sample. Under atmospheric conditions (at around 50 °C, the lowest temperature around 10 °C), only the P-10-MCC coated Cu specimen undergoes corrosion shown by change of its color after two months.

The synthesized 10-MCC PCM must be stored in a suitable container for future use. Hence, different types of materials for containers, for example, Sn (tin), Al (aluminum), stainless steel, galvanized Fe (iron), and Cu (copper) metal, shown in Figure 12, were evaluated. The top surface of the specimens was coated with the 10-MCC PCM, and their surface properties were characterized. The coated metal sheets were exposed to high solar radiation or humid conditions prevailing in Saudi Arabia for 2 months. The appearance, including the color, of Sn, Al, stainless steel, and galvanized Fe in contact with PCM did not change, indicating that these materials are corrosion-resistant and are compatible with 10-MCC. In contrast, Cu specimen coated with 10-MCC PCM showed heavy corrosion after exposure to the same hot and humid conditions (Figure 12). The color of the Cu sheet coated with 10-MCC PCM changed to light blue, indicating that Cu containers are not suitable to store 10-MCC PCM. Weight of the specimens determined before and after exposure using a high-precision analytical balance indicates that all materials except Cu do not undergo any weight loss. Hence, containers made of all tested materials, except the Cu, are suitable to store the P-10-MCC PCM.

### 3.9. Seepage Test

**Figure 13:**
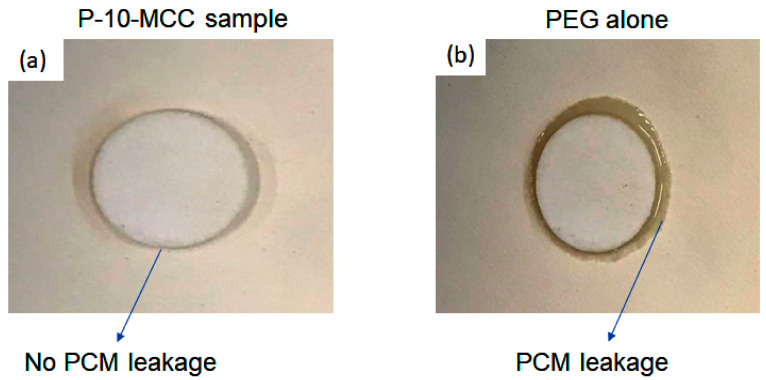
Photographs of (**a**) P-10-MCC (**b**) PEG alone after being heated at 70 °C for checking leakage (seepage test).

Figure 13 shows that the P-10-MCC composite microstructure remains unchanged indicating the absence of leakage (Figure 13a). However, PEG starts to melt after heating to 70 °C (Figure 11b). The tests were performed at 70 °C for 10 min.

### 3.10. Comparison of the Results of the Present Study with Those of Previous Studies

As a support material CaCO_3_ alone is not suitable for encapsulation of PCMs because of the high supercooling. However, as per recently published studies [17,28,45], the encapsulation of PEG-6000 in CaCO_3_ does not reveal the claimed characteristics and/or synergistic properties. Furthermore, literature indicates that the impact of PEG on CaCO_3_ as a PCM has not been explored adequately.

The effect of PEG on CaCO_3_ was recorded by Wang et al. [46] and found that aragonite whiskers can be produced using PEG-20,000. Xu et al. [18] obtained CaCO_3_ aragonite in pure form, similar to JCPDS 05-0453 without encapsulation of PEG. However, the XRD pattern provided in JCPDS 83-0578 was obtained when CaCO_3_ was applied. Additionally, all diffraction peaks are similar to aragonite or calcite, depending entirely on the molecular weight of PEG. The results indicate that CaCO_3_ when mixed with PEG behaves differently. In the present study, a pure calcite phase was obtained via a hydrothermal process without the addition of PEG, which is completely at odds with the results of Xu et al. [18]. Xu et al. [18] obtained needle-like crystals for CaCO_3_ alone, but rhombohedral type crystals were observed for CaCO_3_ with PEG, which are entirely different morphologies.

The synthesis route is a significant factor and in the present study PEG was encapsulated in CaCO_3_ by simply combining PEG-6000 with CaCO_3_. Compared to the present synthesis, which is merely a physical mixing process, when a chemical reaction between the CaCO_3_ matrix and PEG occurs during synthesis different characteristics are obtained. A noteworthy aspect is that the starting phases (XRD patterns) and the synthesis process will decide the chemical or physical properties of the PCMs. Hence, it is important to briefly highlight the recent trends in organic/inorganic microencapsulation—to produce core/shell style PCMs. Microencapsulation suffers primarily from limited application fields, low heat transfer rate, and high microencapsulation process costs [10]. In the conventional microencapsulation method, shells are typically prepared using metals. Wang et al. [28] recently documented a thermal storage system for CaCO_3_ shell/paraffin core system, synthesized by conventional chemical synthesis methods with the addition of surfactants, and demonstrated that a chemical reaction between Ca^2+^ and OH^−1^ is possible when the CaCl_2_ solution is added to paraffin/surfactant aqueous emulsion. The synthesis process is tedious and costly involving chemical reactions, which is in direct contrast to the system proposed in this study. Moreover, Wang et al. [28] did not investigate the supercooling problem and did not provide results for melting and freezing of CaCO_3_. The addition of inorganic material only improves conductivity and leakage problems. Inorganic support does not affect the melting properties PCMs when the melting point is high compared to that of the functional polymer. Yu et al. [19] fabricated a microencapsulated PCM using a CaCO_3_ shell/n-octadecane core with the addition of surfactants. They observed a poor efficiency of encapsulation and a very poor latent heat value compared to that of n-octadecane core (209.10 J/g). Pan et al. [53] have reported that the melting temperature of the microencapsulated PCMs is lower than that of palmitic acid (PA), possibly due to interface interactions between the core and shell of PA@AlOOH. The same authors have also pointed out that in previous studies organic/inorganic compounds have been widely used as the shell, for example silica, and no clear effect on the melting temperature of the microcapsulated system was observed. Thus, the present study used XRD and FTIR for determining the basic physical properties of the PEG-600 and CaCO_3_ mixture. Consequently, the inorganic support should not have any effect except the improvement of thermal conductivity and minimizing the leakage problem. However, the CaCO_3_ support material has a very high phase change temperature of 1300 °C, which does not affect the melting properties of an ss-PCM.

### 3.11. Thermal Conductivity

A quite high conductivity value of 48.00 Wm^−1^ K^−1^ has been reported for MgO [45]. For CaCO_3_ and PEG samples, the conductivity values are 2.167 Wm^−1^ K^−1^ and 0.212 Wm^−1^ K^−1^, respectively [13,39,41]. The thermal conductivity of PEG/10MgCaCO_3_ is 0.5456 Wm^−1^ K^−1^, as listed in Table 2. The conductivity of P-10-MCC is ~62% higher than PEG alone. A low value of thermal conductivity was observed when the molar ratio of CaCO_3_/PEG is higher. A higher molar ratio lowers the content of CaCO_3_. In this study, 0.5:0.2 was used as the PEG:MgCaCO_3_ PCM ratio as it provides the best 69% PEG impregnation in the 10MgO-doped CaCO_3_ (P-10-MCC). The content of CaCO_3_ plays an important role in improving the thermal conductivity. Wang et al. [28] have reported that the CaCO_3_ support enhances the thermal conductivity owing to the higher thermal conductivity of CaCO_3_ itself, most possibly due to its highly compact internal design. The conductivity of PEG/ZSM-5 is also higher, most likely due to ZSM-5 creating new types of thermal conductive routes and/or pathways [42].

Zhang et al. [49] recently published excellent data using Ag-graphene/PEG to boost solar thermal energy conversion and storage. With a thermal conductivity value of 0.414 W/mk, the composite has a high latent heat of 166.1 J/g. The method showed a slightly higher supercooling. This method, however, is not feasible for field applications as graphene is costly.

As this study deals with PEG, stabilized samples based both on paraffin and PEG PCMs were compared owing to their working temperatures being very similar and both are studied for building applications. The results obtained are compared with paraffin-based PCMs in Table 1 and Table 2. Another available form of paraffin (RT 27) is not suitable for actual use because its latent heat and conductivity values are very low at 0.24 W/mk, 54.55 J/g [51]. Karaipekli et al. [50] have reported that ExP/paraffin (n-eicosane (C20) composite PCM can boost conductivity using CNT. For example, the best ExP/C20/CNT sample (1 wt.%) possesses a latent value of 157.43 J/g and a conductivity value of 0.32 W/mK. Lu et al. [52] have studied the paraffin/ExP composite PCM, but the latent heat value is very low. Zhang et al. [54] have recently prepared a shape-stabilized PCM using PEG6000/ExP (ExP = expanded perlite) with an added carbon layer to increase the thermal conductivity of the material and prevent leakage problems. However, a very high quantity of sucrose, i.e., 60%, was used to prepare the carbon layer at high temperature, which is a dangerous method as the sucrose solution can normally trigger an explosion during the drying process. Zhang et al. [54] reported a thermal conductivity of about 0.479 W/mk. More significantly, the conductivity of the carbon layer is not equal to that of CNT. Also, N-eicosane (C20 paraffin) is costlier than PEG.

### 3.12. Solar-to-Thermal Energy Storage Efficiency

The optical properties of the Mg-doped CaCO_3_-PEG PCM, the P-10-MCC allow simultaneous solar-to-thermal energy conversion and thermal energy storage. The Mg-doped CaCO_3_ nanoparticles and/or porous support enhance the visible light absorption of PEG in the entire band and further increase absorption at around ∼300 nm (Figure S1). The enhanced full band absorption and selective absorption will confer excellent photothermal conversion efficiency on the P-10-MCC composite. Based on the UV-vis absorption spectra of their PEG sample and/or Equation (5), Zhang et al. [49] recently determined the solar-to-thermal energy storage efficiency in the visible region for PEG/graphene based PCM.

**Figure 14:**
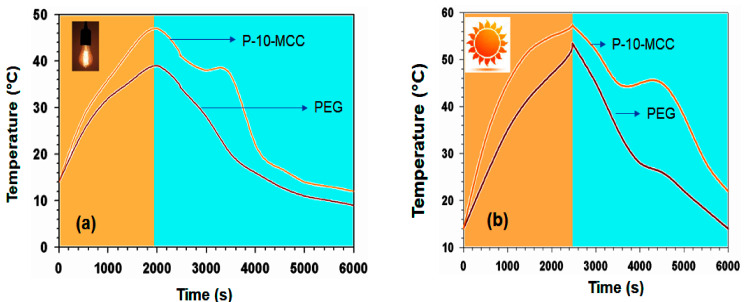
(**a**) Light-to-thermal energy conversion curves of PEG 6000, and P-10-MCC composites under solar simulator irradiation (I = 120 mW cm^−2^). (**b**) Solar-to-thermal energy conversion curves of PEG 6000 and P-10-MCC composite under sunlight irradiation (I = 98 mW cm^−2^, 12:00–14:00, 23 April 2019, Dhahran, Saudi Arabia).

The optical properties of 10-MCC are somewhat higher than that of CaCO_3_ alone. For further confirmation, the UV-vis absorption spectra of PEG alone and P-10-MCC were examined. UV-vis absorption of P-10-MCC is higher than that of PEG alone. Using the present method, solar heat conversion into thermal energy and their energy storage capacity can be calculated simultaneously due to the high latent value and good optical properties [49]. Also, the light absorption spectrum of P-10-MCC was recorded in the entire visible region, which is greater than that of PEG alone. To evaluate the solar-thermal energy conversion capability of P-10-MCC, embedded temperature recorders under solar simulators were used to investigate the solar heat conversion into thermal energy of PEG 6000 and P-10-MCC. Figure 14a indicates an increase in temperature that can be attributed to the high activity of 10-MCC and/or PEG as molecular stoves and photon emission. The temperature of PEG is increased due to infrared light when subjected to solar irradiation. Under long-term exposure to radiation, an optimum value was observed, indicating the storage of thermal energy through a phase change. Due to the release of energy, a cooling stage emerged in the cooling process.

The thermal energy can heat the PCM composite materials and then the energy can be retained in the composites by the PCMs through phase change. The following equation was used to determine solar efficiency in the visible region for thermal energy storage:

(5) $$\eta =\frac{m\Delta H}{\mathrm{IS}\left(T_{t}-T_{f}\right)\;}$$

**Figure 15:**
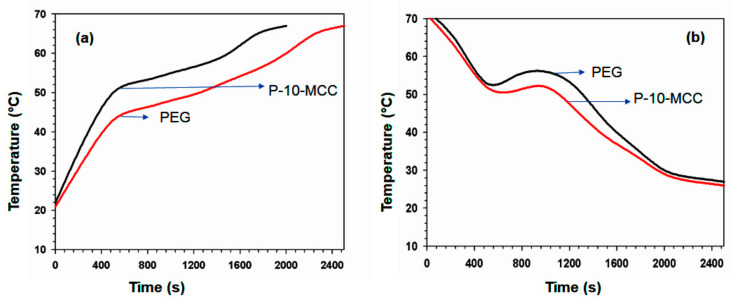
(**a**) Heating temperature curves of PEG 6000 and P-10-MCC composite; (**b**) freezing temperature curves for the same.

The sample weight and the melting phase change enthalpy are denoted by m and ΔH, respectively, while I and S denote the optical power density and the radiated field, respectively, and T_t_ and T_f_ are the transition time points at beginning and ending phase, respectively. The solar-to-thermal energy storage efficiency (η) of P-10-MCC is 62.2%. Compared with reported PCMs containing carbon materials, these findings demonstrate good efficiency in storing photothermal energy. Chen et al. have reported a PCM based on wax impregnated with carbon nanotubes with a thermal storage efficiency of approximately 40–60% [54]. The PEG/10MgCO_3_ PCM was placed under sunlight to evaluate it for actual applications and to determine its performance. Under long term exposure to solar radiation, the temperature increased, and an optimum value was obtained for temperature boosting. An optimum value for decreasing temperature was obtained in the cooling process when the sunlight (Figure 14b) was blocked. This device can convert solar energy into heat energy through phase transformation and is able to store energy. The high enthalpy of P-10-MCC indicates that the produced PCM has a high thermal capacity to meet real world application requirements. Besides, the melting and freezing temperatures in the solar-to-thermal energy conversion curves are compatible with the heating and cooling temperatures on the plateau (Figure 15a,b). After 200 cycles, the solar-to-thermal conversion efficiency is reduced by less than 0.53%.

## 4. Conclusions

Inorganic materials were tested to be used as a support to encapsulate and produce stable PCMs. For the first time a porous material containing a combination of alkaline earth metal ions, i.e., Mg-doped CaCO_3_ was synthesized using a simple hydrothermal process. This method addresses a range of PCM technology issues, including the synthesis of significant amounts of support materials at a low cost using simple techniques. The P-10-MCC PCM has a superior latent heat, larger storage capacity, and low supercooling compared to other composites tested, i.e., P-5-MCC and P-15-MCC PCMs. The P-10-MCC shape-stabilized composite PCM displays reproducible behavior and retains the ability to store and release energy without a significant change even after 200 thermal heating and cooling cycles. The cost of production of shape-selective P-10-MCC PCMs is significantly lower than that of materials prepared using conventional inorganic/organic microencapsulation, as the proposed method uses widely available and low-cost materials (MgO, CaCO_3_, and PEG). P-10-MCC PCMs have a good odor and do not undergo sublimation during melting. Hence, P-10-MCC PCMs may be a potential candidate for ensuring comfort within buildings.

## Figures and Tables

**Table 2:** Thermal conductivity of pure PEG-6000 and PEG-6000/5MgCaCO_3_, PEG-6000/10 MgCaCO_3_, and PEG-6000/15MgCaCO_3_ PCM composites. These values were compared with PEG (P)/ExP (expanded perlite), ExPP-CNT (0.5 wt %), and ExPP-CNT (1 wt %) and other analogous PCMs, i.e., paraffin and Ag/GNS (graphene sheet)/PEG samples.

Material	Thermal Conductivity (Wm^−1^ K^−1^)	Reference
PEG-6000	0.2124	This wok
CaCO_3_	2.167	This work
PEG-6000/5MgCaCO_3_	0.3389	This wok
PEG-6000/10MgCaCO_3_	0.5456	This work
PEG-6000/15MgCaCO_3_	0.5634	This work
PEG6000	0.212	[49]
GNS	0.257	[49]
Ag–GNS/PEG-1	0.317	[49]
Ag–GNS/PEG-2	0.337	[49]
Ag–GNS/PEG-3	0.367	[49]
Ag–GNS/PEG-4	0.414	[49]
Paraffin wax (RT27)	0.166	[50]
RT27/Expanded perlite	0.167	[50]
RT27/EP/Sikalatex(SL)	0.149	[50]
RT27/EP/SL/AL	0.247	[50]
PEG	0.263	[51]
EP	0.058	[51]
PEG/EP	0.161	[51]
PEG/EP/Carbon layer	0.479	[51]
ExP	0.05	[52]
Eicosane (C20)	0.22	[52]
EP/C20 60%	0.15	[52]
EP/C20 60%/CNT 0.3%	0.19	[52]
EP/C20 60%/CNT 0.5%	0.24	[52]
EP/C20 60%/CNT 1%	0.32	[52]

The measured maximal deviations for conductivity values were ±0.05 Wm^−1^ k^−1^ taking into account the averages of the five measurements.

## Data Availability

The data presented in this study are available on request from the corresponding author.

## Supplementary Materials

The following are available online at https://www.mdpi.com/article/10.3390/nano11071639/s1, Figure S1: UV-vis adsorption spectra of PEG and P-10-MCC.
