# Supplementary material for: Preparation of a Sustainable Shape-Stabilized Phase Change Material for Thermal Energy Storage Based on Mg2+-Doped CaCO3/PEG Composites
Source: Nanomaterials (Basel). 2021 Jun 22;11(7):1639. doi: 10.3390/nano11071639 (PMC8306958; doi:10.3390/nano11071639)
Supplement: Supplementary file 1 [file nanomaterials-11-01639-s001.zip › nanomaterials-1212935 supplementary1.pdf]

Preparation of a Sustainable Shape-Stabilized Phase Change Material for Thermal Energy Storage Based on Mg^{2+}-Doped $CaCO_3$ / PEG Composites

Md. Hasan Zahir [1*], Mohammad Mominur Rahman [2], Salem K. S. Basamad [3], Khaled Own Mohaisen[4], Kashif Irshad [1], Mohammad Mizanur Rahman [5], Md Abdul Aziz [6], Aasif Helal[6], Amjad Ali [1], Mohammad M. Hossain [3,7]

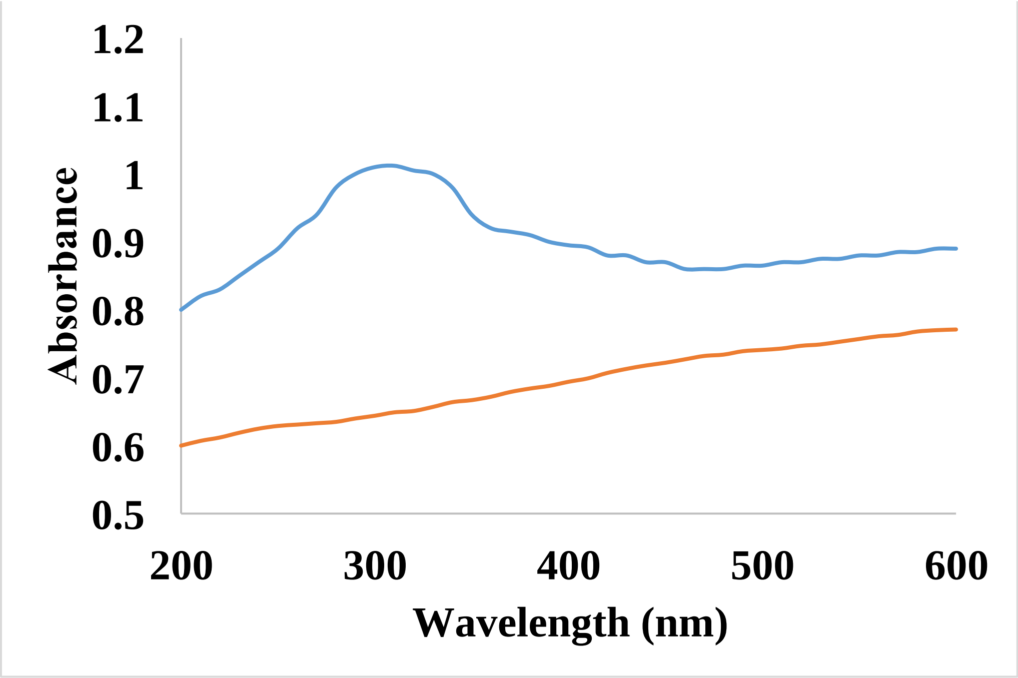

Figure S1. UV-vis adsorption spectra of PEG (pink line) and P-10-MCC (blue line).
